# Beautiful Hair

**Published:** 1877-08

**Authors:** 


**BEAUTIFUL HAIR**—Finds worshipers the world over. When it is abundant and tastefully adjusted, it "sets off" the face of beauty, and may be made to soften even a deformity. Its arrangement exhibits the taste of the wearer, and it may be made more ornamental than the richest jewel ever dug from Golconda's mines. But it is a jewel not found in the casket of one woman in a thousand. The hair should be cultivated from infancy, by keeping the head clean and cool night and day; it should be worn as short as a boy's until the thirteenth year is completed; a comb, or a pin, or a tie should not be allowed for an hour, nor a "parting" nor a braid; nothing should ever touch it but a tortoise or fine rubber-comb and pure soft water; it should never be allowed to bear more than its own weight, nor detained from its natural direction; if any thing, it should be supported unstrained. Ventilation and cleanliness, from infancy to budding womanhood, are essential to having hair that is thick, long, luxuriant, permanent, and glistening with life. Fashion, all blind and remorseless as she is but too often, is for once philosophical and wise in introducing nets to hold the hair of girls at the neck behind.

The pernicious metallic hair-pins have "killed" the hair of our wives and daughters, whose entire stock in trade, that is their own, scarcely equals in size a common hickory-nut. It has been cut by the harsh hair-pin, pulled from its roots by braiding and tying, and actually rotted at its origin by an imbedded mass of grease, dust, and dandruff, stopping up every pore, preventing exhalation, confining the heat, and setting up a permanent and destructive inflammation about every bulb. A clean scalp and pure soft water are the best pomatums in the world for man or woman, boy or girl, young or old.

